# Total parathyroidectomy with trace amounts of parathyroid tissue autotransplantation as the treatment of choice for secondary hyperparathyroidism: a single-center experience

**DOI:** 10.1186/1471-2482-14-26

**Published:** 2014-05-05

**Authors:** Qingqing He, Dayong Zhuang, Luming Zheng, Ziyi Fan, Peng Zhou, Jian Zhu, Songjian Duan, Yanning Li, Yanming Ge, Zhen Lv, Lei Cao

**Affiliations:** 1 Department of Thyroid and Breast Surgery, Jinan Military General Hospital of PLA, No.25 Shifan Road, Jinan 250031, People's Republic of China; 2 Blood Purification Center, Jinan Military General Hospital of PLA, Jinan 250031, People's Republic of China

**Keywords:** Secondary hyperparathyroidism, Chronic renal failure, Total parathyroidectomy, Parathyroid hormone, Autotransplantation

## Abstract

**Background:**

The aim of the study was to evaluate total parathyroidectomy with trace amounts of parathyroid tissue (30 mg) as a surgical option in secondary hyperparathyroidism (sHPT) treatment.

**Methods:**

From January 2008 to March 2012, 47 patients underwent parathyroidectomy. Comparisons of demographic data, symptoms, and preoperative or postoperative biochemistry were made between total parathyroidectomy with trace amounts of parathyroid tissue autotransplantation group and total parathyroidectomy group.

**Results:**

Out of 47 cases, 45 had successful operation. 187 parathyroid glands identified at the initial operation were reported in 47 patients. 43 patients had been diagnosed with parathyroid hyperplasia, and 4 patients had a benign adenoma. After operation, pruritus, bone pain and muscle weakness disappeared, also serum PTH and serum phosphate were declined markedly as well. After discharge, two patients (in total parathyroidectomy group) were readmitted because of postoperative hypoparathyroidism. Graft-dependent recurrence was not observed in an average follow-up of 42 months.

**Conclusions:**

Total parathyroidectomy with sternocleidomastoid muscle trace amounts of parathyroid tissue autotransplantation is considered to be a feasible, safe and effective surgical option for the patients with sHPT.

## Background

SHPT is ubiquitous in patients with chronic kidney failure treated by long-term dialysis. High parathyroid hormone (PTH) levels can lead to 1) renal osteodystrophy, 2) calciphylaxis, 3) ectopic calcifications, 4) abnormal fat and sugar metabolism, 5) refractory pruritis, and 6) anemia [1-3]. New treatments for sHPT include vitamin D analogues and calcimimetics. When vitamin D analogues and calcimimetics fails, parathyroidectomy becomes necessary. Surgical treatment of sHPT may involve various surgical approaches [3-6]. Surgical options for the treatment of sHPT include total parathyroidectomy, subtotal (3 1/2 gland) parathyroidectomy and total parathyroidectomy with heterotopic autotransplantation. However, surgeons have been debating on the optimal operative management for patients with sHPT, and surgical consensus on the optimal procedure has not been reached [7-10]. The aim of this study is to examine our experience with patients who underwent total parathyroidectomy with or without trace amounts of parathyroid tissue autotransplantation for sHPT.

## Methods

### Patient eligibility and study design

**Figure 1:**
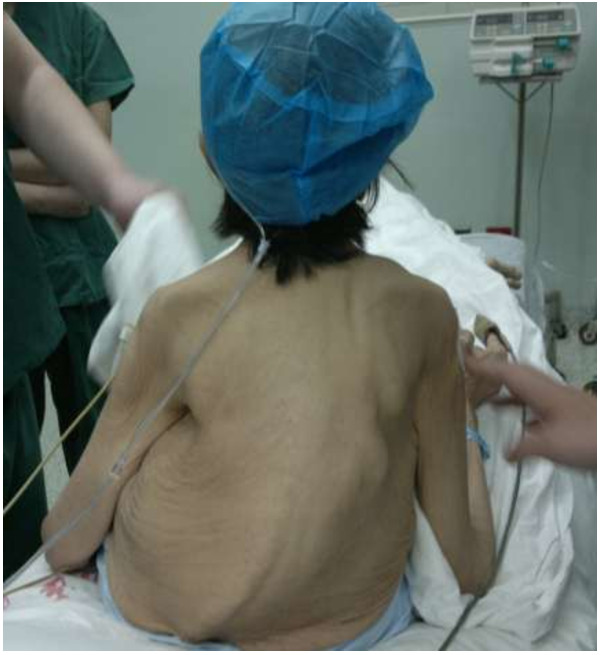
Bone deformities.

We performed a retrospective study of 47 patients with chronic renal failure, who experienced total parathyroidectomy with or without sternocleidomastoid muscle autografting for sHPT from January 2008 to March 2012. There were 26 men and 21 women and their mean age was 46 years old (range, 28–71). Forty-seven patients underwent hemodialysis for an average of 8.9 years (range 3–19 years). Heights dropped an average of 6 cm in all patients. After stosstherapy with Calcitriol 2 μg, twice per week, all symptoms such as pruritus, bony pain, movement disorder, anorexia and kyphosis (Figure 1) were getting progressively worse, with serum PTH and calcium level exceeding the normal range. Our indications for parathyroidectomy in symptomatic patients with sHPT include elevated levels of serum PTH (>1600 ng/L), persistent hypercalcemia, intractable pruritus, osteodystrophy or calciphylaxis, bone pain, and pathological fracture, large-sized parathyroid glands (volume > 1.0 cm^3^) on ultrasonography, and/or hyperphosphataemia (>2.26 mmol/L).

Forty-seven sHPT haemodialysis patients provided written informed consent for the study, and the study was approved by the Jinan Military General Hospital Research Ethics Board. The decision to perform total parathyroidectomy with or without autotransplantation was based on a preoperative thorough discussion with the patient. 14 patients chose total parathyroidectomy with trace amounts of parathyroid tissue autotransplantation. 33 patients, not eligible for kidney transplantation, underwent total parathyroidectomy.

### Preoperative preparation

Forty- six patients showed 1–4 radionuclide dense areas in the neck by ^99m^Tc-MIBI SPECT scan. All cases had routine preoperative tests including complete blood counts, serum electrolytes, chest x-ray, electrocardiogram, and coagulation screening. Surgical intervention in the form of parathyroidectomy is generally considered only in cases of severe sHPT. However, identification of the exact location of the parathyroid glands before parathyroidectomy is challenging. The surgeon attended the ultrasonography examination, which was helpful to get an exact localization of parathyroid glands (by ultrasonography and scintigraphy) and perspicuous understanding of their relationship with the surrounding tissues. 139 hyperplastic parathyroid glands were detected by B-ultrasonography in 47 cases, 2 to 4 enlarged parathyroid glands in each case. Two cases had cervical CT scanning and two and three hyperplastic parathyroid glands were discovered respectively. All cases had hemodialysis three times per week and heparin-free hemodialysis was performed the day before operation. Forty-seven patients underwent intravenous ^99m^Tc-MIBI 2 hours prior to surgery.

### Surgical procedure

All patients were operated under supervision of one single surgeon. With the patients under anesthesia cervical plexus and neck hyperextended, a Kocher's incision is made, preferably in a skin crease. Tissues from skin to muscles were dissected layer by layer until the thyroid gland was exposed. Then the middle thyroid veins were cut off, and the thyroid gland was turned over medially. With the combination of preoperative ultrasonography, intraoperative exploration, and intraoperative gamma probe detection, all of the parathyroid tissues were carefully discovered and removed. The operation was performed carefully avoiding any damage to the parathyroid capsules. Resection of the surrounding fat tissue of original parathyroid glands is essential.

All removed tissues were intraoperatively verified by frozen section. Surgical pathology reports of parathyroid tissues removed were reviewed in all procedures performed at our hospital. The resected tissues were kept in sterilized iced saline. If fewer than four glands were found, then total parathyroidectomy without autotransplantation and transcervical thymectomy were undertaken. Thymectomy was not performed routinely if four parathyroid glands had been confidently identified. Diffuse-type hyperplastic parathyroid tissue(no nodular hyperplastic parathyroid tissue) was chosen for autografting. The estimated weight of the autograft tissue is 30 mg. After the strap muscles were closed, a 1.0 cm pocket was developed parallel to sternocleidomastoid muscle fibers. After it was certain that there was no bleeding in the muscle pocket, 30 mg of parathyroid gland (cutting into pieces of 1 mm^3^ in size) without nodular hyperplasia was implanted into the muscular bed of the patients' sternocleidomastoid muscle. The muscle fibers anterior to the pocket containing the parathyroid tissue were then sutured closed with unabsorbable sutures, and the cephalad and caudad portions of the autograft site were marked with titanic clips for future reference, in case further reduction of functioning parathyroid tissue became necessary. Care was taken not to traumatize the area of the autograft during closure of the incision, in an effort to avert bleeding into the area that causing impaired revascularization of the parathyroid tissue.

Intraoperative PTH was performed trying to confirm total removal of the parathyroid glands, so as to avoid overlooking remaining or supernumerary glands. Peripheral venous blood sample (5.0 mL) was obtained immediately after induction of anesthesia and 30 minutes after removal of all parathyroid glands.

If the surgeon identifies 3 glands and cannot identify the 4th gland, or intraoperative PTH was greater than 400 ng/L, complete cervical exploration of ectopic sites (explore retropharyngeal and esophageal spaces, trace recurrent laryngeal nerve into chest, open carotid sheath, and do not perform median sternotomy) and cervical thymectomy were performed [11,12].

Three patients had undergone thyroidectomy additionally (one patient with papillary thyroid microcarcinoma and 2 cases with diffuse Hashimoto thyroiditis and large multinodular goiter).

Approximately 80 mg of the parathyroid tissue will be placed in cold sterile saline solution (containing a solution of 80% culture media, 10% patient serum and 10% dimethyl sulfoxide) for later cryopreservation. The vial is then cooled to -70°C at a decreasing rate of 1°C per minute.

### Postoperative management

For the prevention of postoperative tetany, all patients received oral or intravenous calcium (higher dialysate Ca levels in 45 patients) if indicated. 45 patients were given calcium supplement postoperatively through a deep vein infusion pump to maintain adequate calcium levels. The serum calcium concentration was monitored every 12 hours to adjust the oral and/or intravenous calcium treatment. Calcium gluconate of 8–16 g per 24 hours was administered for one to two weeks, then, the rate and times of calcium infusion were adjusted according to the results of serum calcium measurement to maintain the serum concentration within the range of 1.8-2.3 mmol/L and finally until the serum calcium was stabilized (usually in one to two weeks). Afterwards, oral calcium administration (calcium carbonate of 3-12 g per day) was prescribed. Meanwhile, postoperative oral Calcitriol was administrated with the dose of 0.25-2.5 μg per day for one to three weeks, gradually diminished to 0.25-0.5 μg per day in total parathyroidectomy with autotransplantation group, and 0.75-1 μg per day in total parathyroidectomy group.

Higher Ca dialysate is helpful to sustain normal serum Ca levels. Parathyroid hormone was measured postoperatively, and heparin-free hemodialysis was resumed for every patient, 24 hours postoperatively.

Patients were discharged when corrected calcium levels were above 2.0 mmol/L and free of hypocalcemic symptoms. Follow-up biochemical markers included serum phosphate, calcium, levels of 25-Hydroxyvitamin D3 (1,25-Hydroxyvitamin D3 is better, but now we are inaccessible of this test, will be included later) and PTH.

After discharge, a follow-up visits at 1, 3, 6, 12 months, and every six months thereafter are executed. Follow-up visits consist of telephone interview, in-clinic visits, direct mail, and email. A brief telephone questionnaire including follow-up medical records and biochemistry studies at local affiliated hemodialysis hospitals was used to evaluate patients’ compliance with postoperative oral calcium, Calcitriol and symptom relief.

### Statistical analysis

The continuous data were tested by the *t* -test, and categorical data were tested by the chi-square test or Fisher’s exact test. The statistical software used was Social Sciences version 15.0. Differences were considered significant at *p-value* <0.05.

## Results

**Figure 2:**
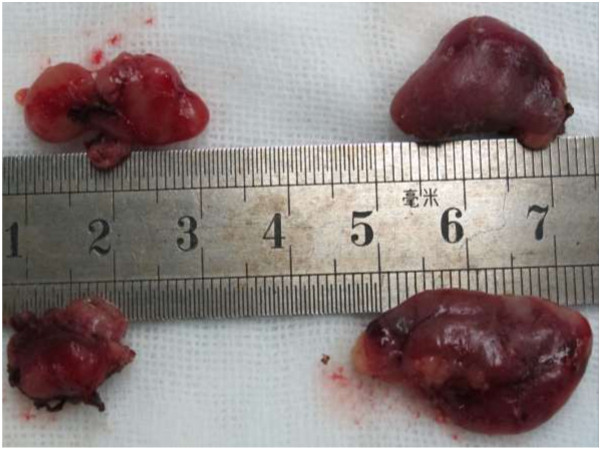
Four parathyroid glands identified at the primary operation in patient for secondary hyperparathyroidism.

**Figure 3:**
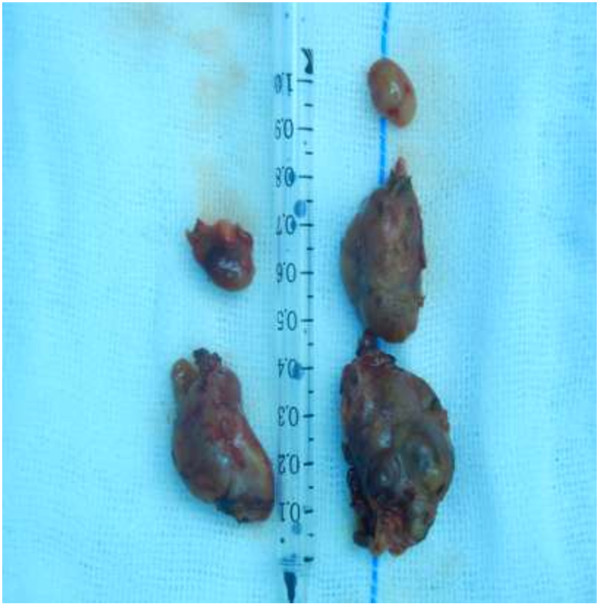
Five parathyroid glands identified at the primary operation in patient for secondary hyperparathyroidism.

One hundred and eighty-seven parathyroid glands identified at the initial operation were reported in 47 patients (Table 1). Thirty-three (35.1%) inferior glands were located in the thymic tongue. 43 of the 47 patients had the diagnosis of parathyroid hyperplasia, four patients had a benign adenoma. 45 patients had 4 parathyroid glands (Figure 2). More than 4 glands were discovered only in one patient at the primary operation (Figure 3). Three glands were removed in 1 patient. After operation, pruritus, bone pain and muscle weakness disappeared, and serum PTH and serum phosphate were declined markedly (Table 2).

**Table 1:** Demographics, symptoms, and preoperative or postoperative biochemistry

**Parameter**	**tPTX+AT Group (n=14)**	**tPTX Group (n=33)**	***P *** **Value**
Age, median (years)	46.5±13.9 (28–63)	45.8±14.2 (29–71)	>0.05
Men/women	8/6	18/15	>0.05
Duration of dialysis (years)	9 (3–19)	8.8 (3–17)	>0.05
Renal transplant (no.)	2	5	>0.05
Follow-up, mean (months)	43.1 ±8.9 (9–62)	41.6±10.4 (9–62)	>0.05
Preoperative BMD (Z-score)	-3.72±1.12	-3.66±0.98	>0.05
Symptoms			
Fractures or bone pain (no.%)	14 (100)	33 (100)	>0.05
Kidney stone (no.%)	0	0	
Pruritus (no.%)	12 (85.7)	30 (90.9)	>0.05
Fatigue/weakness (no.%)	8 (57.1)	20 (60.6)	>0.05
Osteoporosis (no.%)	13 (92.8)	32 (96.9)	>0.05
Preoperative biochemistry			
PTH (ng/L)	2746 ± 886 (2430–3809)	2798 ±983 (1600–4280)	>0.05
Ca (mmol/L)	2.6 ±0.21 (2.28-2.81)	2.58 ±0.23 (2.3-2.95)	>0.05
P (mmol/L)	2.5 ±0.73 (1.75-3.91)	2.52 ±0.82 (1.77-4.31)	>0.05
25(OH)D3(ng/ml)	10.31±2.35	10.45±2.41	>0.05
Postoperative 1st day biochemistry		(n=31)	
PTH (ng/L)	35.3±16.8	16.8±7.5	>0.01
Ca (mmol/L)	1.92 ±0.38	1.79±0.37	>0.05
P (mmol/L)	1.83±0.71	1.76±0.68	>0.05
Symptoms relief (no.%)		(n=31)	
Bone pain (no.%)	14 (100)	31 (100)	<0.05
Pruritus (no.%)	12 (100)	30 (96.8)	>0.05
Postperative BMD (Z-score)	-2.83±1.92	-3.16±1.68	>0.05
Recurrence	0	0	<0.05
Postoperative symptomatic recurrent hypocalcemia	0	2	<0.05
Postoperative 6 months biochemistry		(n=31)	
PTH (ng/L)	13.69±6.29	8.87±6.15	<0.05
Ca (mmol/L)	2.01±0.19	1.91±0.21	<0.05
P (mmol/L)	0.895±0.18	0.95±0.22	<0.05
25(OH)D3 (ng/ml)	16.36±2.67	11.71±2.34	<0.05

*Abbreviations*: *tPTX+AT* total parathyroidectomy with autotransplantation, *tPTX* total parathyroidectomy, *BMD* bone mineral density, *PTH* parathyroid hormone, *25(OH)D3* 25-Hydroxyvitamin D_3_.

**Table 2:** 187 parathyroid glands identified at the primary operation in 47 patients for secondary hyperparathyroidism

**Located in behind the thyroid gland**	**No. of parathyroid glands**	**The size (mm)**	**The volume (mL)**
Right superior parathyroid gland	47	5-22(9.1±4.1)	0.5-2.2(0.9±0.4)
Right inferior parathyroid gland	47	18-35(24±4.8)	1.7-3.6(2.1±0.5)
Left superior parathyroid gland	46	5-20(8.8±3.7)	0.6-2.1(1.1±0.47)
Left inferior parathyroid gland	47	15-33(21±4.9)	1.6-3.5(2.2±0.8)

**Figure 4:**
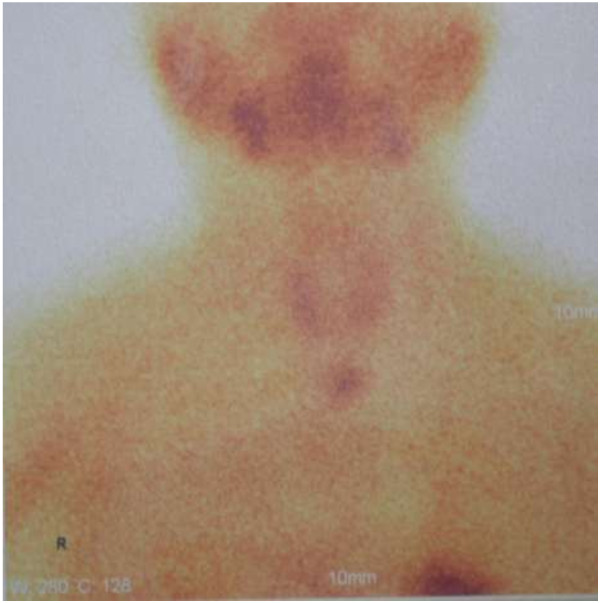
**The 5**^**th **^**parathyroid gland was confirmed by positive sestamibi **^**99 m**^**Tc investigation in the mediastinum in 1 year after the surgery.**

Of 47 cases, 45 had successful operation. 2 patients had persistent hyperparathyroidism in total parathyroidectomy group(one patient was found the 5th supernumerary gland in 1 year after the surgery, Figure 4). The detecting sensitivity combined with preoperative ^99m^Tc-MIBI-SPECT scan, intraoperative gamma probe detection, intraoperative PTH assay, and intraoperative exploration for parathyroid glands was 95.7%. No recurrent laryngeal nerve palsies were encountered. And there were no perioperative deaths. Complications were two cervical bleedings following postoperative hemodialysis which needed surgical intervention.

The duration of hospitalization was 10.5 days on average. Graft-dependent recurrence was not observed. No evidence of clinical bone disease and pathological fractures detected after a mean observation period of 42 months (range: 9–62 months). After discharge, two patients (in total parathyroidectomy group) were readmitted because of postoperative symptomatic recurrent hypocalcemia (PTH:2.39 ng/L and 3.57 ng/L). One patient who underwent kidney transplantation after total parathyroidectomy with trace amounts of parathyroid tissue autotransplantation did not develop serious hypocalcemia or osteomalacia. Rudimentary and split glands were not present in all cases.

## Discussion

Chronic renal failure is a powerful stimulus for parathyroid cell proliferation. With the development of new technologies, the survival time of the haemodialysis patients is being obviously extended. SHPT is a severe and frequent complication in patients with advanced chronic kidney disease, characterized by hyperaplasia of all parathyroid glands and elevated serum PTH levels [13,14]. Parathyroidectomy is only used when all medical therapy (new therapeutic agents including vitamin D-receptor activators and calcium-mimetics, and new phosphate binders) is unsuccessful [15]. In China, the prevalence rate of sHPT patients with parathyroidectomy indication is unknown. Furthermore, in recent years contradictions and disputes of physician-patient are increasingly intensified in China. It is thought that the access of sHPT patients to surgical treatment is limited and particularly restricted to university centers. Parathyroidectomy indication is practically unequivocal. According to our experience, the presence of extraskeletal calcification, calciphylaxis, debilitating bone disease, refractory pruritus, severe hypercalcemia, and PTH >1600 ng/L are strong indications for surgical treatment.

The best surgical approach for renal hyperparathyroidism is yet to be determined and remains controversial in the literature. There are three accepted main types of surgical procedures and variations of these approaches (subtotal parathyroidectomy, total parathyroidectomy, and total parathyroidectomy with autotransplantation) [15,16]. Every procedure has its own disadvantages besides advantages. Sanbury [17] first described subtotal parathyroidectomy in 1960, the major defect of subtotal parathyroidectomy is that it is very difficult to recognize which gland is suitable for preservation by observation with naked eyes. This may cause recurrence of hyperparathyroidism. Once recurrence occurs, re-exploration of the neck is required, thus there is a greater risk of injuring the recurrent laryngeal nerves than at the initial operation, and reoperation also seems to introduce the risk of parathyromatosis.

Total parathyroidectomy and autotransplantation (the smallest gland was chosen) was first performed by Wells in 1975 [18]. Small amounts of resected parathyroid tissue can be autografted in the muscles of the forearm or neck, as well as in the subcutaneous tissue of the chest or abdomen. A randomized study suggested that total parathyroidectomy with autotransplantation had a lower rate of recurrent sHPT and an improved clinical outcome compared to subtotal parathyroidectomy. Reoperation was for persistent sHPT in 82 of 485 (17%). Findings at reoperation included: autograft hyperplasia (49%), supernumerary glands (20%), remnant hyperplasia (17%), a missed in situ gland (7%), and a negative exploration (5%). Reoperation determined that inadequate cervical explorations occurred in 42% of patients who had undergone a subtotal parathyroidectomy and in 34% of patients who had undergone a total parathyroidectomy with autotransplantation [14]. Tominaga who had the biggest series consisting of 2660 patients reported that graft-dependent recurrent sHPT was 248(9.3%), and 216 (8.1%) patients underwent only removal of the autograft and 32 (1.2%) required both removal of the autograft and resection of residual parathyroid gland in the neck or mediastinum [19]. We thought that 30 pieces of 1 × 1 × 3 mm parathyroid tissue was more than 30 mg for autografting. Operative failures occur because of the limitations in preoperative localization, inadequate exploration, and the natural history of hyperplastic parathyroid tissue. Although we were unable to identify the superiority of one operation over the other, operative success can be improved with an adequate cervical exploration. We believe sternocleidomastoid muscle is a better autografting area because of easy accessibility, one operative site, less graft ischemia, low incidence of infection, and high success rate due to excellent blood supply. But, sterno-cleido-mastoid muscle is not the standard site for autotransplantation in secondary hyperparathyroidism. In the recurrent condition, ^99m^Tc-MIBI-SPECT/CT scan(a imaging modality that enabled reliable and precise localization of the parathyroid) is able to distinguish between a cervical recurrence and a graft depending recurrence. Diffuse-type hyperplastic parathyroid tissue preferably was chosen for autografting to prevent recurrent sHPT. Recurrent HPT after total parathyroidectomy with autotransplantation is usually due to overgrowth of the autograft. We consider thirty pieces of 1 × 1 × 3 mm parathyroid tissue (about 100 micrograms) too much. Ten pieces of 1 × 1 × 3 mm parathyroid tissue (about 30 micrograms) is enough. For patients who demand long-term hemodialysis after parathyroidectomy, the risk of recurrence is not negligible. Thus, to avoid recurrent HPT, all parathyroids, including supernumerary glands, should be identified. In our series, 35.1% of inferior glands were located in the thymic tongue. If the surgeon cannot identify 4 glands, removal of thymic tissue from the neck incision is essential to avoid missing glands. Most patients underwent artificial arteriovenous fistula in bilateral forearms for hemodialysis. We believe it would be easier and safer to remove residual parathyroid tissue from sternocleidomastoid muscle at recurrence as compared to from the forearm. Reoperation may be relatively simple for recurrent hyperparathyroidism induced by the hyperfunction of autografted parathyroid tissue, because surgical access to sternocleidomastoid muscle is simple, and it does not require general anesthesia, also avoid the risk of major surgical complications such as laryngeal nerve injury.

In order to suppress parathyroid secretion completely and reduce recurrent hyperparathyroidism, total parathyroidectomy without autotransplantation was performed in 1967 [20]. Patients who had total parathyroidectomy without autotransplantation had temporary hypocalcemia postoperatively and became asymptomatic, despite hypocalcemia, when they were supplemented with calcitriol in the long term. After all types of surgery, attention must be kept on patients' postoperative period. The lack of osteoclastic activity caused by a decrease in PTH postoperatively may lead to a precipitous fall in calcium levels, a condition called “hungry-bone syndrome” [21]. While the autographed parathyroid tissue begins to function in two to three weeks, patients may have severe hungry bone syndrome in this period. It is necessary that all patients received calcium and calcitriol supplement for low calcium levels.

The function of the grafted tissue can be followed in most patients by the determination of the PTH concentration. In 14 of our cases we perform trace amounts of parathyroid tissue (30 mg) autotransplantation and in follow-up (9–62 months) no recurrence observed. The sPTH level has been used to evaluate the results of parathyroidectomy. Ideally, the completeness of the operation could be biochemically confirmed by utilizing the intraoperative PTH assay. If intraoperative PTH is more than 400 ng/L, supernumerary parathyroid gland may not be completely resected. The extent of operative exploration for supernumerary glands may become less controversial with widespread utilization of the intraoperative PTH assay as an adjunct in guiding the completeness of resection. The utilization of intraoperative PTH monitoring may become a useful adjunct for assuring that the patient has undergone an adequate parathyroidectomy in sHPT [22-26]. Patients whose PTH level dropped under 400 ng/L by the removal of parathyroid gland, sHPT could be controlled by medical treatment after the operation. Patients who underwent total parathyroidectomy without autotransplantationusually had almost untraceable sPTH levels immediately after surgery, but later it can be detected again. The rise in serum PTH may be a result of parathyroid cell re-proliferation stimulated by hypocalcemia in these patients. A similar biochemical rebounding tendency was observed. In our study, clinical symptom relief has no statistical difference between total parathyroidectomy with trace amounts of parathyroid tissue autotransplantation group and total parathyroidectomy group. Bone pain disappeared in almost all patients within 1 week after surgery. Total parathyroidectomy without autotransplantation leads to harmful definitive hypoparathyroidism condition. Clinical experience showed that total parathyroidectom had disadvantages, such as candidate for kidney transplantation, lifelong substitution therapy, and osteopenia developed in the absence of PTH [6,15,26,27].

## Conclusions

The total parathyroidectomy with trace amounts of parathyroid tissue autotransplantation is a feasible, safe and effective surgical option for the patients with sHPT.

## Competing interests

The authors declare that they have no competing interests.

## Authors' contributions

QH had operated cases and analyzed all data. DZ, LZ and ZF participated in the design of the study and performed the statistical analysis. PZ, JZ, SD,YL, YG, ZL,and LC did the assistant of the operation. All authors read and approved the final manuscript.

## Pre-publication history

The pre-publication history for this paper can be accessed here:

http://www.biomedcentral.com/1471-2482/14/26/prepub

## Statement

Corresponding author had obtained permission from the copyright holder to reproduce the figure, and confirmed that consent to publish the Figure 1 by the patient.
